# IGF-1 treatment causes unique transcriptional response in neurons from individuals with idiopathic autism

**DOI:** 10.1186/s13229-020-00359-w

**Published:** 2020-06-26

**Authors:** Sara B. Linker, Ana P. D. Mendes, Maria C. Marchetto

**Affiliations:** 1 The Salk Institute, Laboratory of Genetics, La Jolla, CA 92037 USA; 2 Department of Anthropology, University of California, San Diego, La Jolla, CA 92037 USA

**Keywords:** Induced pluripotent stem cells (iPSC), Insulin-like growth factor 1 (IGF-1), Disease modeling, Autism

## Abstract

**Background:**

Research evidence accumulated in the past years in both rodent and human models for autism spectrum disorders (ASD) have established insulin-like growth factor 1 (IGF-1) as one of the most promising ASD therapeutic interventions to date. ASD is phenotypically and etiologically heterogeneous, making it challenging to uncover the underlying genetic and cellular pathophysiology of the condition; and to efficiently design drugs with widespread clinical benefits. While IGF-1 effects have been comprehensively studied in the literature, how IGF-1 activity may lead to therapeutic recovery in the ASD context is still largely unknown.

**Methods:**

In this study, we used a previously characterized neuronal population derived from induced pluripotent stem cells (iPSC) from neurotypical controls and idiopathic ASD individuals to study the transcriptional signature of acutely and chronically IGF-1-treated cells.

**Results:**

We present a comprehensive list of differentially regulated genes and molecular interactions resulting from IGF-1 exposure in developing neurons from controls and ASD individuals. Our results indicate that IGF-1 treatment has a different impact on neurons from ASD patients compared to controls. Response to IGF-1 treatment in neurons derived from ASD patients was heterogeneous and correlated with IGF-1 receptor expression, indicating that IGF-1 response may have responder and non-responder distinctions across cohorts of ASD patients. Our results suggest that caution should be used when predicting the effect of IGF-1 treatment on ASD patients using neurotypical controls. Instead, IGF-1 response should be studied in the context of ASD patients’ neural cells.

**Limitations:**

The limitation of our study is that our cohort of eight sporadic ASD individuals is comorbid with macrocephaly in childhood. Future studies will address weather downstream transcriptional response of IGF-1 is comparable in non-macrocephalic ASD cohorts.

**Conclusions:**

The results presented in this study provide an important resource for researchers in the ASD field and underscore the necessity of using ASD patient lines to explore ASD neuronal-specific responses to drugs such as IGF-1. This study further helps to identify candidate pathways and targets for effective clinical intervention and may help to inform clinical trials in the future.

## Background

Autism spectrum disorders (ASD) comprise a group of complex neurodevelopmental disorders that affect one in 68 children in the USA [1]. The current diagnostic criterion characterizes ASD as showing persistent deficits in social communication and interaction and restricted or repetitive patterns of behavior, interests, or activities, with symptoms arising before the age of 3 years. Currently, there are no pharmacological interventions that target the core symptoms of ASD, despite great efforts in the field. Evidence from genetic screening suggests that susceptibility to ASD may arise from mutations in hundreds of different genes [2, 3]. While this heterogeneity has posed a challenge for the development of effective drugs for ASD, recent studies on genetic and transcriptional profiling from both syndromic (monogenic) and non-syndromic (idiopathic) forms of ASD seem to converge on common pathways that are involved in neurogenesis, synaptic development, and chromatin remodeling [2–7].

Insulin-like growth factor 1 (IGF-1) has emerged as a potential treatment option for both syndromic and non-syndromic forms of ASD [4, 8, 9]. A growing body of evidence accumulated in the past years in both rodent [10–14] and human [15–17] ASD models have established IGF-1 as one of the most promising ASD therapeutic interventions to date. The collective evidence for the potential efficacy of IGF-1 in the treatment of ASD core symptoms has encouraged the scientific and clinical community to launch a number of clinical trials for both syndromic and non-syndromic forms of the disorder [18, 19] (ClinicalTrials.gov Identifiers: NCT01525901, for Phelan-McDermid Syndrome; NCT01253317, NCT01777542, for Rett Syndrome; NCT01970345, for Autism Spectrum Disorder).

IGF-1 is a neurotrophic factor that is critical for proper development of the central nervous system (CNS) and plays important roles during neuronal growth, synaptogenesis, survival, and migration [8, 20]. Its effects on early CNS development and neuronal plasticity as well as the ubiquitous presence of its receptor, IGF1R, in the adult brain suggest that IGF-1 can act as an endocrine, paracrine, and autocrine hormone [8, 21]. Under normal conditions, IGF-1 exerts its actions by binding to its receptor (IGF1R) and activating two main pathways, PI3K/AKT/mTOR and MAPK/ERK [8, 22], that have been previously implicated in ASD [22]. Furthermore, these pathways have important downstream effects on transcription of key factors involved in synaptogenesis, synaptic transmission and maintenance, and neuronal plasticity such as presynaptic protein synapsin 1 (Syn-1) and post-synaptic density protein-95 (PSD-95) [20, 23–25]. Indeed, deficits in synaptic function and plasticity in glutamate signaling have been consistently documented in syndromic and non-syndromic mouse and human neuronal models of ASD and IGF-1 treatment has rescued the deficits in the same models [10–15, 17, 26]. While IGF-1 interacts with pathways that are implicated in ASD core pathology (e.g., PI3K/AKT/mTOR and MAPK/ERK) and its administration has proven effective in reversing the phenotypic and neuronal functional changes in ASD mouse and human models, little is known about the specific downstream targets of IGF-1 treatment in the context of ASD cellular pathology.

Induced pluripotent stem cells (iPSCs) constitute an ideal model for understanding complex diseases with strong genetic component such as ASD because it allows for the study of unique aspects of patients’ neuronal development in vitro. Here, we used a previously characterized, neuronal population derived from iPSC from neurotypical and idiopathic ASD individuals to study the transcriptional signature of acutely or chronically IGF-1-treated cells. Our results indicate that in neurons derived from neurotypical individuals, IGF-1 treatment altered the gene expression profile in pathways previously identified as downstream of IGF-1 receptor (IGF1R) signaling such as the MAPK pathway. Conversely, response to IGF-1 treatment in neurons derived from ASD patients was heterogeneous, indicating that IGF-1 response may have responder and non-responder distinctions across large cohorts of ASD patients and that the response is correlated with IGF1R expression and spontaneous neuronal activity. Lastly, we show that both chronic and acute IGF-1 treatment recovered a subset of genes significantly enriched within synaptic activity that were involved in baseline transcriptional differences between ASD and controls.

The results presented in this study provide an important resource for researchers in the ASD field and underscore the necessity of using ASD patient lines to explore ASD neuronal-specific responses to drugs such as IGF-1. This study further helps to identify candidate pathways and targets for effective clinical intervention and may help to inform clinical trials in the future.

## Results

### IGF-1 treatment impacts transcription in neurons derived from neurotypical patients

**Fig. 1:**
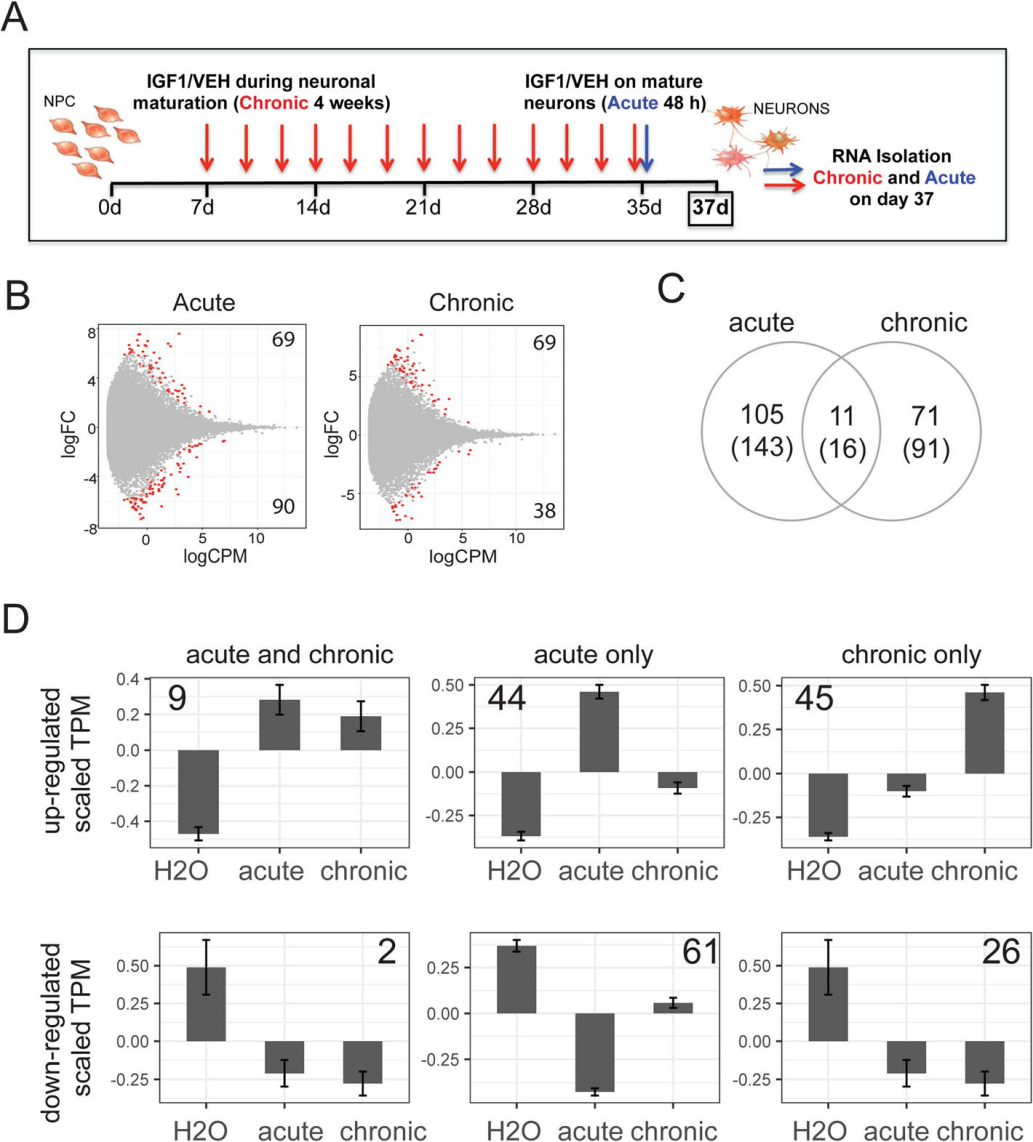
Transcriptional response to IGF-1 in iPSC-derived neurons from neurotypical individuals. **a** Schematics of differentiation of ASD and control cells highlighting the chronic and acute timeline for recombinant human IGF-1 treatment and subsequent RNA isolation. **b** MA-plot of differential expression between neurons from neurotypical control individuals incubated either in media with the addition of water or after exposure to acute (left) or chronic (right) IGF-1. *logCPM* log counts per million, *logFC* log fold-change. Red dots = genes p-adj < 0.05, grey dots = genes p-adj > 0.05. Numbers in the top and bottom corners indicate the number of transcripts differentially expressed with a logFC > 0 or logFC < 0. **c** Overlap in genes (transcripts) identified as differentially expressed with p-adj < 0.05 in the acute and chronic conditions. **d** Expression patterns of differentially expressed genes that were shared between acute and chronic conditions (left), only identified in the acute condition (middle), or only identified in the chronic condition (right). Y = TPM values scaled by gene. Number in corner = number of genes in each group

We first assessed the effect of IGF-1 treatment on iPSC-derived neurons from neurotypical controls using a patient cohort previously described in Marchetto et al. 2017 (Supplementary Table 1) [16]. To understand the molecular changes that are a consequence of IGF-1 treatment, we performed RNA-sequencing on neurons derived from seven neurotypical individuals (two replicates each) treated either with water, or an acute (48-h) or chronic (28-day) dose of IGF-1 solubilized in water (treatment scheme on Fig. 1a). We first excluded outliers based on gene count and position in principal component space which resulted in 13, 14, and 14 samples for the water, acute IGF-1, and chronic IGF-1 treatments respectfully (Supplementary Figure 1A-B). Cell type signature analysis identified neuronal genes as the most highly expressed in all samples followed by astrocyte signatures. As a negative control for the computational cell type model, we examined the prevalence of microglial markers, which would not be expected to be present at high levels in the neuronal preparation. As expected, microglial markers exhibited low detection in all samples with no differences between ASD and neurotypical control (CTL). We also observed no differences in cell type-specific transcriptional signatures between ASD and CTL samples for oligodendrocytes (microglia *p* = 0.95, oligodendrocyte *p* = 0.58, astrocyte *p* = 0.51, neuron *p* = 0.115) (Supplementary Figure 1C). We have also provided fluorescent-activated cell sorting data for PSA-NCAM during neuronal differentiation and we did not observe significant differences in the percentages of PSA-NCAM-positive neurons in controls or ASD (Supplementary Figure 1D). Differential expression analysis with the edgeR generalized linear model method and controlling for patient id (expression ~ treatment + patient) identified 116 genes (159 transcripts) and 82 genes (107 transcripts) differentially expressed (false discovery rate (FDR) = 0.05) in the acute or chronic condition respectively versus water (Fig. 1b, c, Supplementary Table 2). To determine the significance of the magnitude of the transcriptional response to IGF-1 treatment, we calculated the number of genes that passed our FDR cutoff after 100 random permutations of the treatment labels. We calculated bootstrap significance estimates of *p* = 0.01 for acute and *p* = 0.31 for chronic samples indicating that the magnitude of the acute signature was robustly associated with treatment while the magnitude of the chronic signature was not different from random chance (Supplementary Figure 2A-B). To further determine the robustness of the specific genes, rather than the overall magnitude of transcriptional change, associated with IGF-1 treatment, we built a classification algorithm using the random forest design [27] with the significant genes as the features. Both models based on acute and chronic IGF-1 exposure had low error rates (acute = 3.7%, AUC = 0.95; chronic = 0%, AUC = 1.00) (Supplementary Figure 2A-B). Together, these results indicated that IGF-1 altered the gene expression profile in neurons derived from neurotypical individuals and that the acute IGF-1 treatment had a more robust effect on transcriptional change.

When analyzing the acute and chronic conditions together, we identified 105 and 71 genes that were specifically differentially expressed in the acute and chronic conditions respectively, and 11 genes (16 transcripts) that overlapped between the two conditions (*N*_genes total_ = 21231, Fisher exact test *p* < 8.07e-13, OR = 31.03, 95% CI = 14.3–61.06) (Fig. 1c). To understand the progression of transcriptional change of time, the average temporal patterns of expression were visualized for all 187 genes found to be significant in either group (Fig. 1d). Genes that were significant in both acute and chronic conditions, on average, had the same magnitude of change from the baseline water condition indicating that these genes represented a sensitive and constant response to IGF-1 treatment (Fig. 1d, left panels). The genes that were consistently regulated by IGF-1 treatment (nine genes up: *ETV1*, *GPS2*, *HSP90AB1*, *KCTD2*, *PABPN1*, *PRKAR1A*, *PSMD8*, *RAD23A*, and *SPG20*; two genes down: *SCMH1* and *TUBA1A*) consisted primarily of chromatin remodelers, as well as transcription factors, a potassium channel, and multiple genes related to MAPK signaling, indicating a potential for IGF-1 to have large downstream impacts on the cell state through altered chromatin and MAPK signaling dynamics. Interestingly, many of these genes have been associated with ASD and other neurodevelopmental disorders indicating that caution may be warranted when using IGF-1 on neurotypical controls.

We identified 44 genes that were upregulated and 61 genes that were downregulated only in the acute state. Upregulated genes were enriched for the MAPK cascade-involved factors and IGG1-set, and downregulated genes enriched in actin binding and genes important for cell size (Fig. 1d, middle panels). Transcripts which were only significant in the acute state showed a slow return to baseline levels; genes that were induced by IGF-1 returned to baseline more rapidly than genes that were suppressed by IGF-1 (*F* test acute vs. H_2_O *p* = 2.2e-16, chronic vs. H_2_O *p* = 0.13; *F* test acute vs. H_2_O *p* < 2.2e-16, chronic vs. H_2_O *p* < 1.07e-13). Together, this indicated that many differentially expressed genes identified only in the acute condition retained low level effects in the chronic state.

We further identified 45 upregulated and 26 downregulated genes that passed FDR only in the chronic condition. Similar to our findings above, even though these genes did not pass FDR in the acute state, the cumulative signature showed significant magnitude changes in the acute state compared to H_2_O in both the increased (*F* test acute vs. H_2_O *p* < 1.42e-03, chronic vs. H_2_O *p* < 2.2e-16) and decreased (*F* test acute vs. H_2_O *p* < 3.26e-07, chronic vs. H_2_O *p* < 2.2e-16) gene categories, indicating that the chronic effect likely slowly develops over time with small effects present at the acute timepoint (Fig. 1d, right panels). The genes that increased with chronic IGF-1 treatment were largely associated with cell cycle and the genes that decreased in the chronic condition were enriched for kinases and also associated with DNA binding. Together, these results suggest that there is a dynamic wave of transcriptional change occurring in iPS-derived neurons following IGF-1 treatment with separate transcriptional events occurring in the acute and chronic phases. Additionally, the transcriptional change observed is consistent with other reports in the literature indicating that the IGF-1 acts by binding to its receptor (IGF1R) and activating MAPK/ERK signaling pathway [8].

### The transcriptional impact of IGF-1 is unique in ASD-derived neurons

**Fig. 2:**
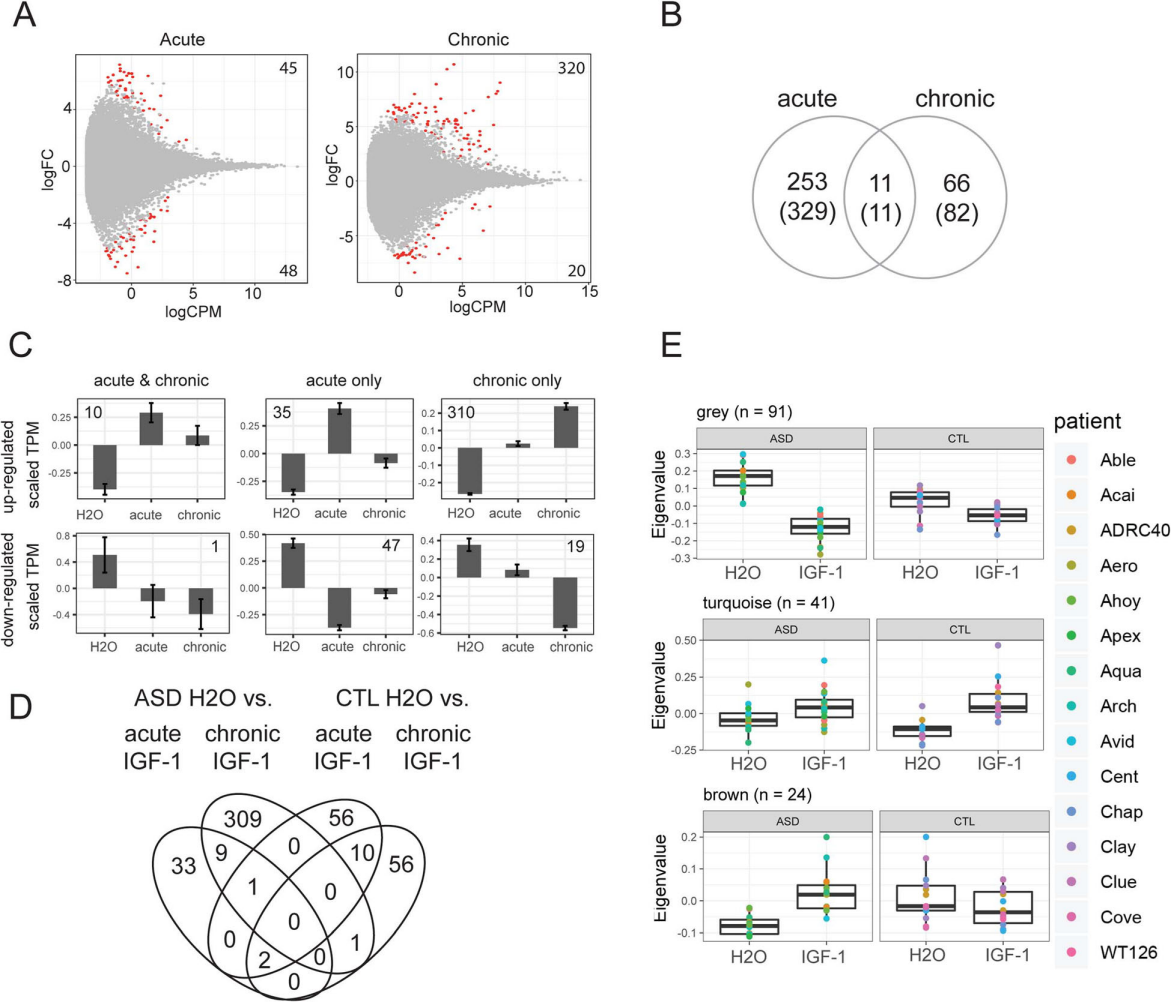
Transcriptional response to IGF-1 in iPSC-derived neurons from ASD individuals. **a** MA-plot of differential expression between neurons from ASD individuals incubated either in media with the addition of water or after exposure to acute (left) or chronic (right) IGF-1. *logCPM* log counts per million, *logFC* log fold-change. Red dots = genes p-adj < 0.05, grey dots = genes p-adj > 0.05. Numbers in the top and bottom corners indicate the number of transcripts differentially expressed with a logFC > 0 or logFC < 0. **b** Overlap in genes (transcripts) identified as differentially expressed with p-adj < 0.05 in the acute and chronic conditions. **c** Expression patterns of differentially expressed genes that were shared between acute and chronic conditions (left), only identified in the acute condition (middle), or only identified in the chronic condition (right). Y = TPM values scaled by gene. Number in corner = number of genes in each group. **d** Overlap between genes differentially expressed between ASD H2O and IGF-1 (acute or chronic) and genes that are differentially expressed between neurotypical controls (CTL) in H2O versus IGF-1 (acute or chronic). **e** Eigenvalue expression within WGCNA gene modules (grey, turquoise, and brown) that were significantly associated with the interaction of disease and IGF-1. *n* = gene count within each module

To examine the effect of IGF-1 in ASD neurons, differential expression analysis was performed on patients diagnosed with ASD (eight patients, two replicates each) with 15, 15, and 13 samples remaining after outlier detection for water, acute IGF-1, and chronic IGF-1 treatments respectively. Further, 78 genes (93 transcripts) were differentially expressed between acute IGF-1 treatment and H_2_O and 265 genes (340 transcripts) between chronic IGF-1 treatment and H_2_O (Fig. 2a, Supplementary Table 2). The acute IGF-1 gene list had power to determine IGF-1 treatment with an error of 3.3% (AUC = 1.00) with a lower estimate when using chronic genes; error 21.43% (AUC = 0.85). Both the acute (bootstrap *p* = 0.56) and chronic (bootstrap *p* = 0.15) conditions did not pass significance using permutation analysis indicating that while the magnitude of differential expression in ASD neurons was not different from random expectation, the individual genes were robustly associated with IGF-1 treatment (Supplementary Figure 2C-D). Further, 253 genes (329 transcripts) and 66 genes (82 transcripts) were uniquely expressed in the acute and chronic conditions, respectively, with 11 genes (*ACTG1*, *CCT7*, *GLYR1*, *NCOA7*, *RACGAP1*, *RTN3*, *SNRPN*, *STK40*, *TSKU*, and *TXLNA*) shared between both exposures (*N*_genes total_ = 21489, Fisher exact test *p* < 2.2e-16, OR = 152, 95% CI = 51.4–500.9) (Fig. 2b). The dynamics of the acute and chronic responses were similar to the observations in neurotypical controls where IGF-1 altered gene expression showed a deviation at both the acute and chronic time points in all gene groups, indicating that acute and chronic IGF-1 gene signatures share a large degree of overlap (Fig. 2c). Interestingly, in comparison to the effect in controls, ASD neurons showed a higher enrichment of genes that displayed changes in the chronic state indicating that ASD neurons may have a slower dynamic change in response to IGF-1 than controls (Pearson’s Chi-squared test *X*^2^ = 92.3, df = 2, *p* value < 9.05e-21; Figs. 1c and 2c). Overall, IGF-1 induced transcriptional changes showed very little overlap between ASD and controls with only four genes overlapping (*CCT7*, *CFL1*, *FTH1*, and *SNRPN;* Fig. 2d). We next asked whether the overall patterns of transcriptional change shared similarity between ASD and controls in the context of acute IGF-1 exposure. An unsupervised WGCNA analysis using all genes revealed low power that was unable to detect gene modules, supporting that the gene expression changes induced by IGF-1 were relatively subtle. Therefore, to directly assess the shared transcriptional changes induced by IGF-1 in ASD and control, we ran a co-expression analysis with a supervised WGCNA using the genes identified as differentially expressed in either the ASD or control samples. We identified four modules (blue *n* = 27, brown *n* = 24, grey *n* = 91, and turquoise *n* = 41) with three of the four modules significantly associated with IGF-1 treatment (grey *F* test *p* < 3.6e-16, brown *p* < 1.4 e-03, turquoise *p* < 3.7e-03) and the same three groups associated with treatment as an interaction with disease status (grey *F* test *p* < 3.9e-07, brown *p* < 6.3e-03, turquoise *p* < 4.8e-02). The largest signature, grey, was that of genes which were strongly altered by acute IGF-1 exposure in ASD (Fig. 2e, top panel). Interestingly, in controls, the same set of genes generated eigenvalues that, in response to IGF-1, shifted in the same direction as IGF-1-treated ASD samples indicating that the genes which were differentially expressed in ASD were also modulated to a lesser degree in controls. A similar effect was observed in the second largest module, turquoise (Fig. 2e, middle panel). The final module of genes, brown, were genes that were differentially expressed as a function of acute IGF-1 primarily in ASD (Fig. 2e, bottom panel). Together, these results identified that the transcriptional response to IGF-1 is sensitive to the disease status of the individual. Importantly, we did not identified any SNVs or CNVs [16] within IGF-1 pathway genes as annotated by the PANTHER database, indicating that the differences in expression in these ASD patients were unlikely due to direct genetic effects from within the IGF-1 pathway (Supplementary Table 1).

### The acute IGF-1 effect from controls is present heterogeneously in ASD samples in association with IGF-1 receptor (IGFR)

**Fig. 3:**
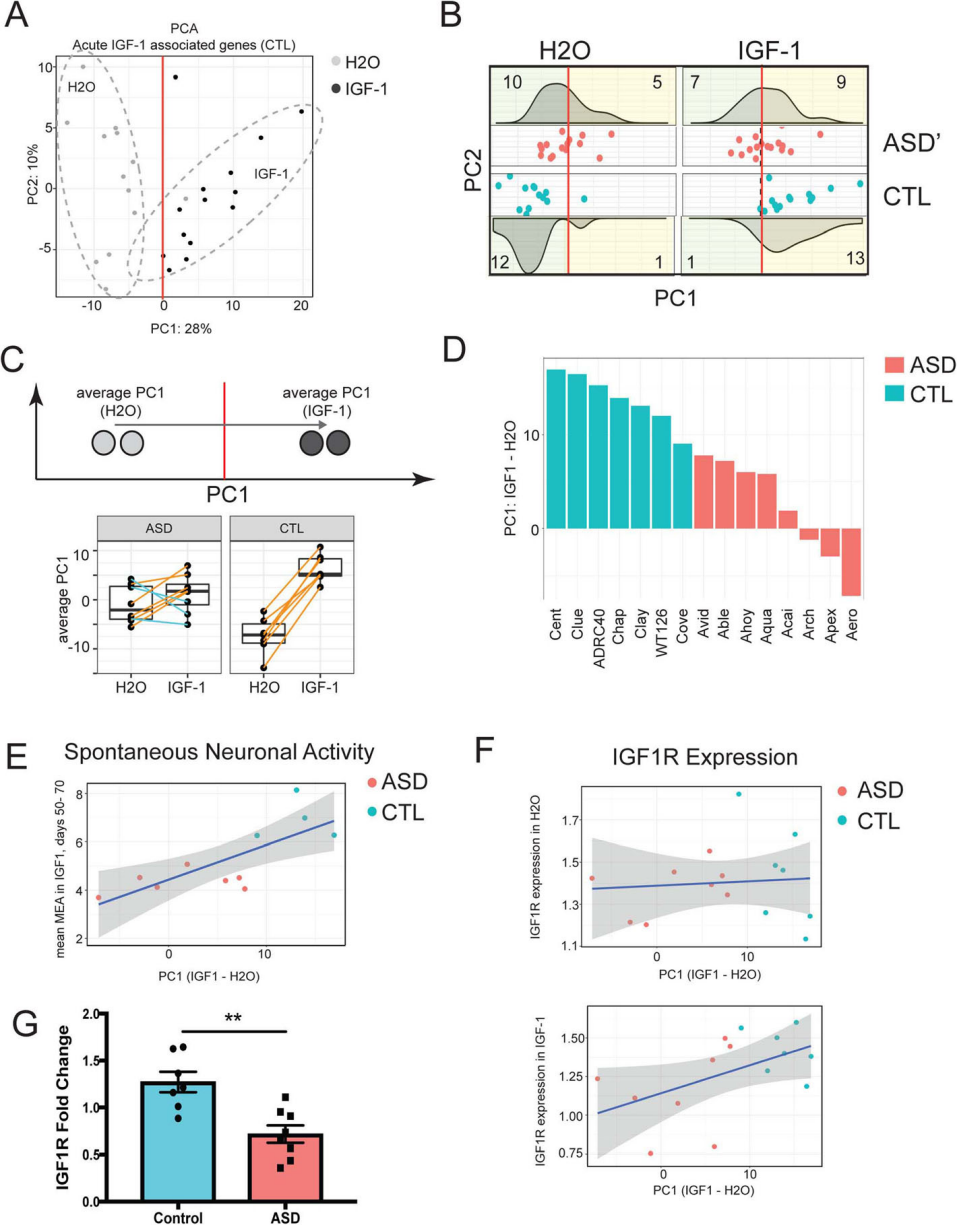
Quantification of the heterogeneous transcriptional response to IGF-1. **a** Principal component plot derived from neurotypical controls (CTL) either in baseline (H2O) conditions or treated acutely with IGF-1. Differentially expressed genes associated with acute IGF-1 treatment were used to generate PC axes that clearly separate H_2_O from IGF-1 and which can be used to assess additional datasets. **b** ASD samples either from baseline (H_2_O) or treated acutely with IGF-1 were projected onto the PC space calculated in (**a**) (ASD’). Each scatter plot inset is plotted along the same PC1 and PC2 axes as in (**a**) and are separated by sample condition (ASD-H2O, ASD-IGF-1, CTL-H2O, or CTL-IGF-1). pink dots = H2O, blue dots = acute IGF-1. The density of dots along the *x*-axis for each condition is plot as density plots above or below each scatter plot. The number of samples on the left or on the right side of PC1 = 0 are noted in the corners of the corresponding density plots. **c** The average PC1 value was calculated for each sample in H2O or after acute IGF-1 treatment and these values are plotted as boxplots. Each dot represents a sample with lines connecting the sample between the average PC1 value in H2O and the value after acute IGF-1 treatment. Samples which shifted to the right along PC1 are connected by a line colored in orange while samples that shifted to the left along PC1 are colored in blue. **d** The difference between the average PC1 value in acute IGF-1 and in H_2_O is plotted as a bar plot for each sample. Blue = CTL, red = ASD. **e** Correlation between the shift along PC1 from H_2_O to acute IGF-1 versus mean spontaneous neuronal activity as previously measured by multielectrode array (MEA) after IGF-1 treatment starting on day 37 [16]. Blue line = least squares regression fit, grey = confidence interval. Red dots = ASD samples, blue dots = CTL samples. **f** Correlation between the shift along PC1 and IGF1R expression either before (top) or after (bottom) IGF-1 treatment. Blue line = least squares regression fit, grey = confidence interval. Red dots = ASD samples, blue dots = CTL samples. **g** Quantitative RT-PCR validating IGF1R differential expression in ASD samples compared to CTL after IGF-1 treatment, *p* = 0.0037, Mann-Whitney *U*

The co-expression analysis finding that the effect of IGF-1 was shared to a small level between ASD and control samples indicated that the response to IGF-1 was heterogeneous. Therefore, to better understand the transcriptional impact of IGF-1, we next sought to quantify and understand what factors may be drivers of that heterogeneity. We focused analysis on the control acute IGF-1 signature, which we showed above was the gene set that was significant in both the permutation test and random forest classification analysis indicating that the transcriptional signature was highly robust. PCA of control samples using this gene set as input separated H_2_O and IGF-1-treated samples on PC1 (*F* test *p* < 2.8e-07) (Fig. 3a). We then used PC1 as an axis of IGF-1 response which could be used to examine the control-like response to IGF-1 in the ASD samples. To accomplish this, we projected ASD H_2_O and acute IGF-1 samples onto the same PC space (Fig. 3b). We then calculated the shift of each patient along PC1 from the position in H_2_O to the position of that same sample in IGF-1 (Fig. 3c). All control individuals had a positive shift along PC1 with similar levels across patients (Fig. 3c, d). Interestingly, we also identified a positive, though reduced, increase for five of the eight ASD samples, which further supported the co-expression results that the acute control-IGF-1 signature was present, to a smaller degree, in ASD neurons (Fig. 3c, d). Importantly, we further identified three ASD samples with a negative shift along PC1 highlighting the variability of this response across patients and indicating that IGF-1 response may have responder and non-responder distinctions across large cohorts of ASD patients (Fig. 3c, d).

To determine if this measure of heterogeneity had physiological relevance to the cell lines, we next compared the shift along PC1 to the electrophysiological activity that we had measured previously in these same neurons after IGF-1 treatment using multiwell electrode arrays [16]. Strikingly, we identified that PC1 was highly associated with mean spontaneous bursts after IGF-1 treatment (*F* test *p* < 7.1e-03; Fig. 3e) indicating that the PC1 measure of heterogeneity was relevant to the electrophysiological characteristics of the patient-derived neurons. Further analysis versus gene expression data identified that the shift along PC1 was also significantly associated with IGFR-1 receptor (IGF1R) levels after acute IGF-1 treatment (*F* test *p* < 0.05; Fig. 3f bottom panel) but not IGF1R levels in water (*F* test *p* = 0.77; Fig. 3f top panel) indicating that the response of the IGF-1 receptor after IGF-1 treatment was associated with the degree of change in the overall transcriptional response to IGF-1 treatment and that this shift was weaker in ASD samples than in controls. IGF1R was detected as differentially expressed between ASD and controls via RNA-seq (padj < 0.05). To validate that IGF1R was differentially expressed in ASD samples after acute IGF-1 treatment, we performed quantitative RT-PCR on RNA from neurons derived from ASD and controls (CTL) treated with IGF-1. We showed that IGF1R is expressed to a lower level in ASD samples compared to controls (*p* < 0.005) confirming our observations by RNA-seq (Fig. 3g, Supplementary Figure 1E).

### IGF-1 treatment recovers ASD-specific expression profiles

**Fig. 4:**
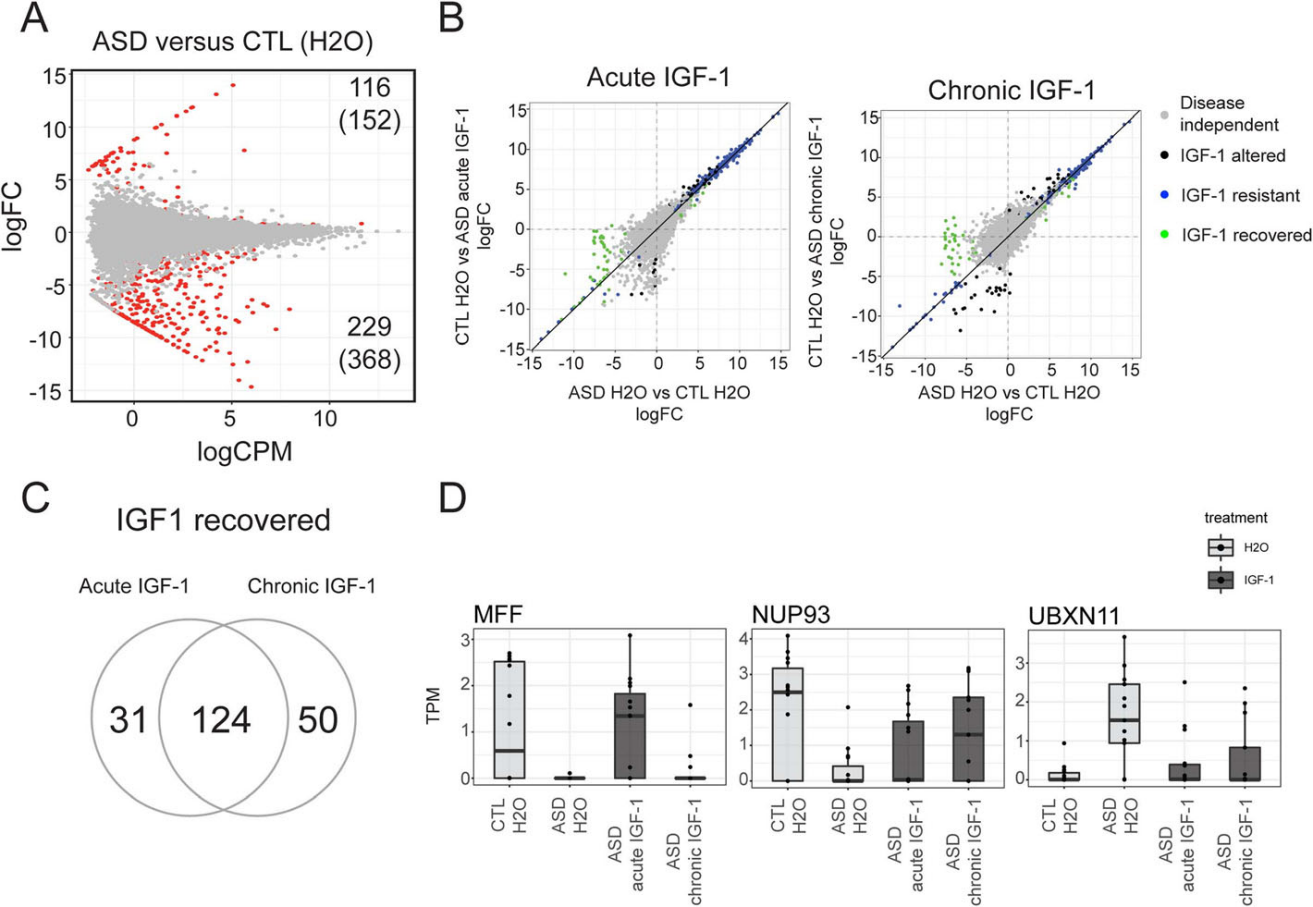
Recovery of a subset of ASD-associated expression patterns after treatment with IGF-1. **a** MA-plot of differential expression between neurons derived from either neurotypical controls (CTL) or ASD individuals both in baseline (H_2_O) conditions. *logCPM* log counts per million, *logFC* log fold-change. Red dots = genes p-adj < 0.05, grey dots = genes p-adj > 0.05. Numbers in the top and bottom corners indicate the number of genes (transcripts) differentially expressed with a logFC > 0 (higher expression in controls) or logFC < 0 (higher expression in ASD). **b** Correlation plot of the logFC between ASD and CTL (*x*-axis) and the logFC between ASD samples in H_2_O and after acute (top) or chronic (bottom) IGF-1 treatment. **c** Gene overlap between acute and chronic conditions for genes that are recovered by IGF-1 treatment as plotted in panel **b**. **d** Top genes recovered by IGF-1 acute and/or chronic treatment

Lastly, we sought to determine if the impact of IGF-1 treatment on ASD-neurons could rescue any of the baseline transcriptional differences between ASD and controls. Differential expression analysis between ASD-H_2_O and control-H_2_O samples identified 345 differentially expressed genes (520 transcripts) (Fig. 4a). Further, 155 (192 transcripts) and 174 (219 transcripts) genes were shifted in ASD samples by IGF-1 treatment to a signature that was more similar to neurotypical controls indicating recovery after treatment with IGF-1 (Fig. 4b). One hundred twenty-four genes were recovered by both acute and chronic treatments (Fig. 4c, Supplementary Table 3, Supplementary Figure 2A). These genes were significantly enriched within synapse genes (p-adj < 0.03) (Supplementary Table 3) indicating that both acute and chronic treatment of ASD neurons with IGF-1 can recover the expression of a subset of the ASD signature to the state of controls. A further 104 (122 transcripts) and 109 (127 transcripts) were significantly different from baseline only after IGF-1 acute or chronic treatment respectively, termed IGF-1 altered. Interestingly, a subset of ASD-associated genes that were recovered after IGF-1 treatment has previously been linked to neurological disorders such as MFF, a mitochondrial gene that is associated with Leigh-like encephalopathy [28], and Nup93, a nucleoporin important in neuronal differentiation [29] (Fig. 4d). Together, these results indicated that a subset of the ASD-associated transcriptional signature can be recovered to control-like levels by both acute and chronic IGF-1 treatments.

## Discussion

Evidence accumulated in the past years in both rodent [10–14] and human [15–17] ASD models have established IGF-1 as one of the most promising ASD therapeutic interventions to date. In addition, phase I human clinical trials have indicated that recombinant human IGF-1 therapy is safe, well-tolerated, crosses the blood-brain barrier, and preliminary assessment of efficacy showed improvement in some neurobehavioral parameters in syndromic ASD [18].

While IGF-1-treatment has been explored in preclinical and clinical trials for syndromic and non-syndromic ASD with positive outcomes, the molecular mechanisms underlying the neuronal functional recovery in ASD are not well understood. The success of a potential use of IGF-1 molecule as a therapeutic agent for ASD pathology will require a better understanding of its mechanism of action in the context of the specific cell targets (e.g., neural niche) and disease environment (ASD).

Here, we used transcriptomics to understand the downstream molecular changes occurring in patient-derived neurons after acute and chronic exposure to IGF-1. We found that IGF-1 has a robust impact on transcription in both control and ASD-derived neurons, and that the differentially expressed gene lists were largely non-overlapping between these two groups. This disease-associated impact should be considered in future clinical studies where IGF-1 treatment is examined only in non-ASD individuals. While the differentially expressed gene lists were non-overlapping between ASD and control, we found that these same gene sets showed small effects in the reciprocal group. For example, genes that only passed FDR after acute IGF-1 treatment in control did exhibit changes in association with IGF-1 in ASD samples when the gene set was aggregated into a group. Furthermore, the degree of response to IGF-1 was heterogeneous and associated with IGF1R levels following IGF-1 exposure. This is an important distinction given that overall IGF1R levels were significantly lower in ASD compared to control following IGF-1 treatment. These results indicate that there could be a stronger negative feedback onto the IGF1R pathway in ASD. Therefore, future studies should consider the response of the IGF-1 receptor when attempting to treat patients with IGF-1. Furthermore, three of the eight patients had low and even an inverse effect in response to IGF-1, indicating that treatment of large ASD cohorts may result in a split of patients that do and do not respond to IGF-1 treatment. Importantly, the idiopathic cohort used in this study was comorbid with macrocephaly during early postnatal development [16]. Future studies will address weather downstream transcriptional response of IGF-1 is comparable in non-macrocephalic ASD cohorts and if IGF1R expression levels are also decreased. Drug response is a common issue in polygenic heterogeneous conditions and has been well documented for neuropsychiatric disorders such as antidepressant response of serotonin reuptake inhibitors in patients with major depression.

Through these analyses, we also identified gene sets that were impacted by IGF-1 treatment. Interestingly, many of the genes that were impacted by IGF-1 treatment in controls have been associated with ASD and other neurodevelopmental disorders which may indicate that caution should be used when studying the effects of IGF-1 on neurotypical controls. For example, *GPS2* is a component of the nuclear receptor co-receptor (NCOR-SMRT) complex which interacts with methyl CpG binding protein 2 (MeCP2) to regulate silencing and has been implicated in the etiology of Rett syndrome [30], *HSP90AB1* is elevated in ASD patients [31], and the potassium channel protein, *KCND2* (Kv4.2), has been associated with a case of twins that were comorbid for ASD and seizures [32, 33].

Importantly, IGF-1 treatment on ASD-neurons recovered the expression of a subset of genes that were differentially expressed at baseline (vehicle treatment) between ASD and controls. These genes recovered their expression profiles in ASD to a neurotypical level and a subset was significantly enriched within synapse genes that have been previously implicated in ASD such as *SYN1*, *CBLN1*, *ACHE*, *GABRB3*, and *NCS1* [34–38]. Further understanding of the role of these genes in the recovery of ASD neuronal synaptic function may improve our understanding of mechanism for IGF-1 therapeutic properties.

## Conclusion

In conclusion, here, we present a comprehensive list of differentially regulated genes and molecular interactions that will generate invaluable resource for information about specific pathways targeted by IGF-1 in the context of ASD patients’ neural cells. It is our expectation that this study will help informing other researchers and clinicians in the field and contribute to improve basic knowledge and designing of future clinical trials.

## Methods

### Cell lines

For this study, we used a previously characterized, neuronal population derived from iPSC from neurotypical and idiopathic ASD individuals to study the transcriptional signature of acutely or chronically IGF-1-treated cells. The idiopathic ASD patients presented in this study are comorbid with macrocephaly and were previously described in Marchetto et al. 2017 (Supplementary Table 1) [16].

### Neuronal differentiation and IGF-1 treatment

ASD and control neural progenitor lines were differentiated for 37 days as previously described and treated with 20 ng/ml of the recombinant human (rh) IGF-1 protein or vehicle (water) (Fig. 1a). For chronic treatment, we added IGF-1 on day 7 for the following 4 weeks. For acute treatment, we added IGF-1 for 48 h on day 35. We differentiated neural progenitor lines (NPCs) from eight ASD and seven control individuals in duplicates, using methods previously described by our group and others [26]. Briefly, embryoid bodies were generated from iPSCs in non-adherent tissue plates (2 weeks) and plated on adherent plates in the presence of dorsomorphin. Visible rosettes form within 1 week, and are manually picked and cultured in neural progenitor cell medium with FGF2 (DMEM/F12 supplemented with B27 and N2 factors). For neuronal differentiation, NPCs will be dissociated with accutase enzyme and plated at low density in neural differentiation medium (DMEM/F12 supplemented with B27 and N2 factors) containing BDNF, GDNF, cyclic-AMP, and ascorbic acid. Confirmation of the functional rescue response to IGF-1 treatment was tested in parallel with multielectrode array activity (MEA) assessment. All media changes and assays were performed at the same time for all samples.

### Library preparation and RNA-sequencing

RNA was prepped for sequencing using Illumina TruSeq Stranded mRNA Library Prep Kit and sequenced on the Illumina HiSeq 2500 (v4) for single-end 50 bp reads at the Salk Institute Next Generation Sequencing Core. All reads were first analyzed by fastQC and then trimmed for low-quality bases with SolexaQA++ dynamictrim, retaining at least 30 bp [39]. Reads were then pseudoaligned to GRCh38 using kallisto version 0.44.0 [40]. Abundance estimates and TPM estimates were extracted from kallisto and TPM values were log_2_+1 normalized for visualization and dimensionality reduction. Samples were then assessed for quality by examining the total reads aligned and with outlier detection via principal component analysis.

### Cell-type signature analysis

To quantify the proportion of cells corresponding to different cell types in the culture for each sample, we have performed an analysis examining broad cell types (microglia, oligodendroctyes, astrocytes, and neurons) using a dataset provided by McKenzie et al. (2018 #244). Using a regression model (scaled expression ~ disease), we compared the total scaled expression of the top marker genes for each cell type.

### Fluorescent-activated cell sorting for cell quantification

PSA-NCAM-positive neurons were sorted by flow cytometry at 16 days post-differentiation. Prior to sorting, the differentiating neurons were stained with anti-PSA-NCAM-APC (antibody, Miltenyi Biotec).

### Differential expression analysis

All differential expression analyses were performed on raw abundance estimates using either the exact or GLM function from edgeR [41]. The tests are described in context, but in brief, exact tests were performed when only one variable was being assessed and GLM test were performed when calculating an additive or interaction model. In both cases, genes were only considered when at least three samples expressed the gene above a value of 1. For exact tests, we first normalized the raw abundance estimates with the calcNormFactors function. We then estimated common, tagwise, and trended dispersions using the estimateCommonDisp, estimateTagwiseDisp, and the estimateTrendedDisp functions respectively. After running the exactTest function, we then calculated adjusted *p* values using the p.adjust function in R with the fdr method. For glm models, we first designed a variable matrix with the fuction model.matrix in R. We then normalized the raw abundances using the calcNormFactors function and estimated common and trended dispersion using the estimate GLMCommonDisp and estimateGLMTrendedDisp functions respectively. The fit was performed with glmQLFit followed by a calculation of significance with glmLRT. *P* values were adjusted with the p.adjust function using the fdr method.

### Quantitative RT PCR

Total cellular RNA was extracted from 3–5 × 10^6^ cells using the RNA-BEE (QIAGEN), according to the manufacturer’s instructions, and reverse transcribed using the high-capacity cDNA synthesis kit from AB Biosystems. qPCR was done using SYBR green (Life Technologies). qPCR results were analyzed using SDS Software v 3.2 for 7900HT real-time PCR system. Primers used: IGF1R-fw: GTTGGGAAGGGGATCATTTT and IGF1R-rev: CATGAAAACCATTGGCTGTG.

### Multielectrode array analysis for neuronal activity

NPCs of each line were plated at a density of 10,000 cells/well in six wells of a 96-well multielectrode array activity (MEA) plate coated with poly-l-ornithine and laminin for neuronal differentiation as described before [16]. Recordings were performed in a Maestro MEA system and AxIS software (Axion Biosystems) using a bandwidth with a filter for 200 Hz to 3 kHz cutoff frequencies. Spike detection was performed using an adaptive threshold set to 5.5 times the standard deviation of the estimated noise on each electrode. Each plate was acclimatized for 10 min in the Maestro Instrument and recorded for 10 min for quantification. Recordings were performed before media change. Multi-electrode data analysis was performed using the Axion Biosystems Neural Metrics Tool and the mean spontaneous neuronal bursts (spontaneous neuronal activity) value was determined in each well. Averages of wells with at least one active electrode were considered for the analysis presented per each cell line. An electrode was considered active at a threshold of five spikes/min. An average of 14–16 electrodes were active per line, with some variations. Only the wells that exhibited activity were included in the analysis. Neuronal firing synchrony per well was not evaluated for these experiments.

### Permutation tests and random forest analysis

For permutation analyses, samples were randomly assigned to a treatment variable then differential expression tests were performed as above using edgeR GLM function (expression ~ treatment + patient + treatment × patient) with the treatment term explicitly tested for significance after false discovery correction using the R p.value function (method = fdr). Genes were filtered out from the analysis if they were expressed less than six across a sum of all sample. One hundred permutations were performed per analysis. *P* values were calculated as the number of permuted label tests that returned equal or more genes than the correctly assigned labels. For random forest analysis, TPM values from genes identified as differentially expressed with an FDR < 0.05 in the respective test were used as input into the classifier. Random forest analysis was performed using the randomForest package [27]. The confusion matrices were used to assess the strength of the model.

### Gene expression network analysis

Weighted gene co-expression network analysis was performed using the WGCNA package in R [42]. TPM values for all water and acute IGF-1 samples were used as input with outliers excluded, as described above. Genes were restricted to differentially expressed genes in acute conditions for either ASD or neurotypical controls with an FDR < 0.05. The network was constructed using the minimal power required to reach a *R*^2^ scale-free topology model fit > 0.9 (power = 4) using the blockwiseModules function with a maxBlockSize = 10,000, network type = unsigned, TOMType = unsigned, minModuleSize = 10, and reassignThreshold = 0. Eigengene values for each module and each sample were calculated with the moduleEigengenes function and were then correlated to sample variables with the function lm(eigengene ~ disease status + treatment + disease status × treatment). Modules that were significant (*p* < 0.05) for the interaction term were reported in the results section.

### Principal components analysis projection

Principal components were calculated using the pcaMethods package in R [43] using TPM values for genes identified as differentially expressed between neurotypical controls at baseline or after acute IGF-1 treatment. ASD sample were then projected onto this PC space using the function predict. To calculate the shift along PC1 from water to acute IGF-1 treatment, average scores for each patient were calculated within a treatment group.

## Supplementary information

**Additional file 1: Supplementary Figure 1.** Sample outlier exclusion and quantification of cell proportions. (A) Histogram of gene counts for all samples. Samples with log(gene counts) less than 6 were excluded from downstream analysis. (B) PCA of all samples colored by gene count. Outliers by gene count were similarly outliers by PCA. (C) Quantification of the proportion of cells corresponding to different cell types in the culture for each sample. As expected, the highest expression was identified in neuronal genes followed by astrocytes. Importantly, there were no differences of expression of the cell type markers detected between ASD and control (microglia p = 0.95, oligodendrocyte p = 0.58, astrocyte p = 0.51, neuron p = 0.115). (D) Fluorescent activated cell sorting (FACS) data for PSANCAM (a marker for glutamatergic progenitors) showing no significant differences in the percentages of PSANCAM positive neurons in ASD or CTL. (E) Quantitative RT-PCR showing IGF1R differential expression in each ASD cell line and compared to each control (CTL) after IGF-1 treatment.

**Additional file 2: Supplementary Figure 2.** Permutation and random forest analysis of IGF-1 associated genes. (A-D: left panels) Histogram of permutation results indicating the number of genes identified as differentially expressed after randomly permuting IGF-1 treatment labels for controls after acute IGF-1 treatment (A), controls after chronic IGF-1 treatment (B), ASD samples after acute IGF-1 treatment (C), and ASD samples after chronic IGF-1 treatment (D). The red line indicates the number of genes identified in the analysis with the true treatment labels and is marked with the respective bootstrapped p-value. (A-D: right panels). Confusion matrix after random forest classification of IGF-1 status from differentially expressed genes for controls after acute IGF-1 treatment (A), controls after chronic IGF-1 treatment (B), ASD samples after acute IGF-1 treatment (C), and ASD samples after chronic IGF-1 treatment (D). Numbers indicate the number of samples identified in each category.

**Additional file 3: Supplementary Table 1.**

**Additional file 4: Supplementary Table 2.**

**Additional file 5: Supplementary Table 3.**

## Data Availability

The datasets obtained and/or analyzed during the current study will be deposited on the public functional genomics data repository, GEO and will be available from the corresponding author on reasonable request. This study utilizes an existing cohort of human induced pluripotent stem cells (hiPSCs) derived from ASD patients and matched controls. These lines are available to the scientific community and currently banked at the NIMH Stem Cell Center at the Rutgers University Cell and DNA Repository (RUCDR).

## Abbreviations

ASD: Autism spectrum disorder
IGF-1: Insulin-like growth factor 1
IGF1R: Insulin-like growth factor 1 receptor
CTL: Neurotypical control
CNS: Central nervous system
NCOR-SMRT: Nuclear receptor co-repressor complex
MeCP2: Methyl CpG binding protein 2
FDR: False discovery rate
MAPK: Mitogen-activated protein kinase
ERK: Extracellular signal-regulated kinase
PI3K: Phosphoinositide 3-kinase
AKT: Adenylate kinase 3
mTOR: Mammalian target of rapamycin
