# Supplementary figures and images for: IGF-1 treatment causes unique transcriptional response in neurons from individuals with idiopathic autism
Source: Mol Autism. 2020 Jun 26;11:55. doi: 10.1186/s13229-020-00359-w (PMC7320548; doi:10.1186/s13229-020-00359-w)

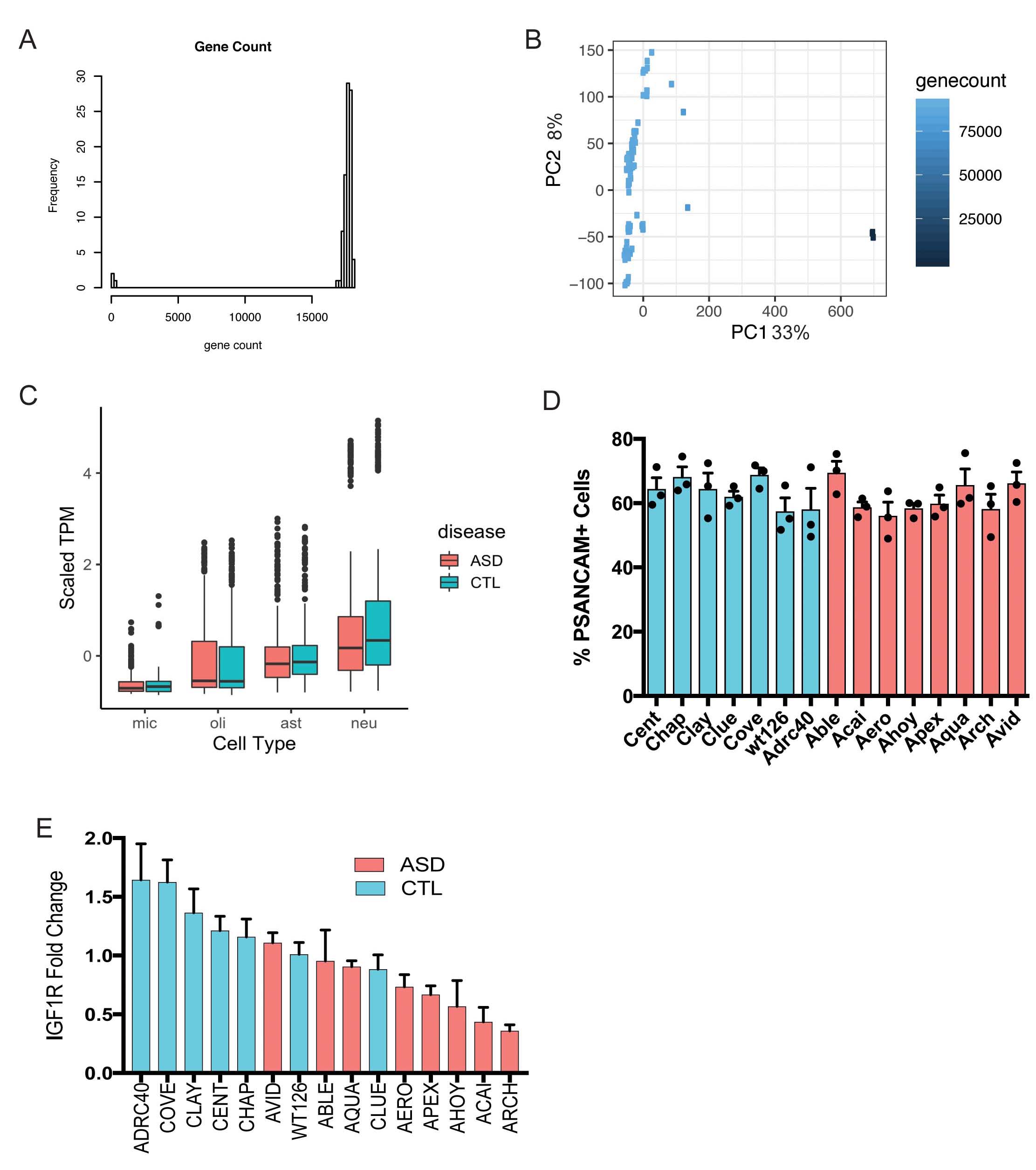

Supplement: Supplementary file 1 — Additional file 1: Supplementary Figure 1. Sample outlier exclusion and quantification of cell proportions. (A) Histogram of gene counts for all samples. Samples with log(gene counts) less than 6 were excluded from downstream analysis. (B) PCA of all samples colored by gene count. Outliers by gene count were similarly outliers by PCA. (C) Quantification of the proportion of cells corresponding to different cell types in the culture for each sample. As expected, the highest expression was identified in neuronal genes followed by astrocytes. Importantly, there were no differences of expression of the cell type markers detected between ASD and control (microglia p = 0.95, oligodendrocyte p = 0.58, astrocyte p = 0.51, neuron p = 0.115). (D) Fluorescent activated cell sorting (FACS) data for PSANCAM (a marker for glutamatergic progenitors) showing no significant differences in the percentages of PSANCAM positive neurons in ASD or CTL. (E) Quantitative RT-PCR showing IGF1R differential expression in each ASD cell line and compared to each control (CTL) after IGF-1 treatment. [file 13229_2020_359_MOESM1_ESM.jpg]

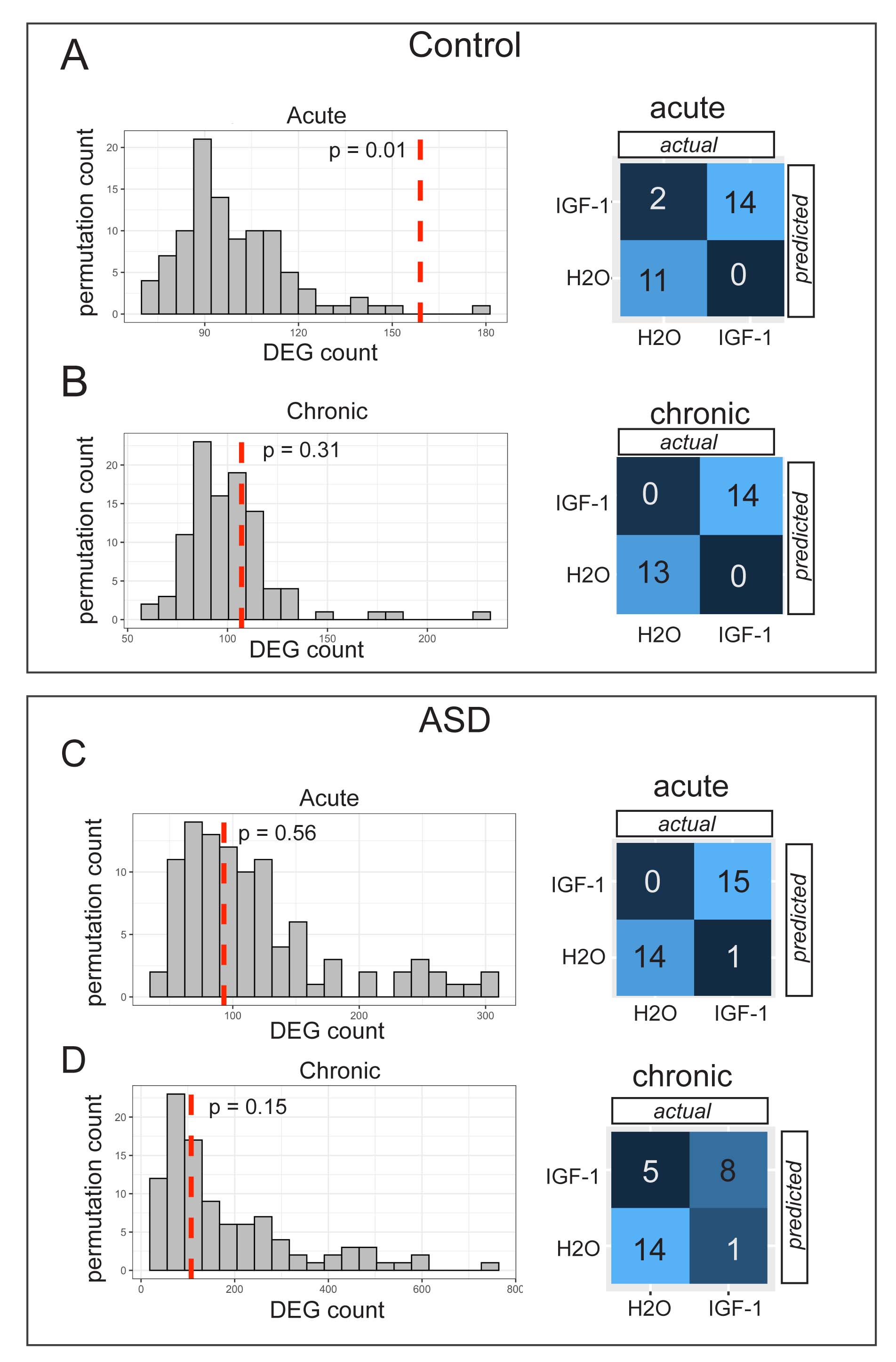

Supplement: Supplementary file 2 — Additional file 2: Supplementary Figure 2. Permutation and random forest analysis of IGF-1 associated genes. (A-D: left panels) Histogram of permutation results indicating the number of genes identified as differentially expressed after randomly permuting IGF-1 treatment labels for controls after acute IGF-1 treatment (A), controls after chronic IGF-1 treatment (B), ASD samples after acute IGF-1 treatment (C), and ASD samples after chronic IGF-1 treatment (D). The red line indicates the number of genes identified in the analysis with the true treatment labels and is marked with the respective bootstrapped p-value. (A-D: right panels). Confusion matrix after random forest classification of IGF-1 status from differentially expressed genes for controls after acute IGF-1 treatment (A), controls after chronic IGF-1 treatment (B), ASD samples after acute IGF-1 treatment (C), and ASD samples after chronic IGF-1 treatment (D). Numbers indicate the number of samples identified in each category. [file 13229_2020_359_MOESM2_ESM.jpg]
